# Ventral hippocampal–postcentral gyrus functional connectivity mediates the association of APOE ε4 gene dose, depressive symptoms, and cognitive function in mild cognitive impairment with subsyndromal depression

**DOI:** 10.3389/fnagi.2026.1843523

**Published:** 2026-07-22

**Authors:** Yang Du, Song Li, Yan Wen, Biao Du, Guoqing Jiang

**Affiliations:** 1 Center for Sleep Medicine, Chongqing Mental Health Center, Chongqing, China; 2 Department of Pharmacy, The Affiliated Three Gorges Hospital of Chongqing University, Chongqing, China; 3 Department of Children and Adolescents, Chongqing Mental Health Center, Chongqing, China

**Keywords:** APOE ε4 allele, functional connectivity, mediating effect, mild cognitive impairment, subsyndromal depression

## Abstract

**Background:**

The apolipoprotein E (APOE) +4 allele is a major genetic risk factor for mild cognitive impairment (MCI) and is associated with hippocampal dysfunction. However, its effects on hippocampal subregional functional connectivity (FC) and clinical manifestations in MCI patients with subsyndromal depression (MCID) remain unclear.

**Methods:**

In this study, 88 patients with MCID were stratified into APOE+4 homozygotes (*n* = 12), heterozygotes (*n* = 31), and non-carriers (*n* = 45). Resting-state FC of the dorsal (dHIP) and ventral hippocampus (vHIP) was examined. Mediation analyses were conducted to investigate the effect of neurological biomarkers on APOE +4 gene dose, depressive symptoms with cognitive function in MCID.

**Results:**

Compared with non-carriers, APOE +4 homozygotes showed decreased FC between the left vHIP and the left inferior temporal gyrus and right precentral gyrus, while heterozygotes showed decreased FC between the left vHIP and the right middle frontal gyrus and left cerebellum. In addition, APOE +4 homozygotes exhibited a gene dose-dependent reduction in FC between the vHIP and the left postcentral gyrus (PoCG) compared with heterozygotes. Importantly, vHIP-PoCG FC mediated the relationship between depressive symptoms and cognitive impairment, and between APOE +4 gene dose and cognitive impairment in MCID patients.

**Conclusion:**

These findings suggest that reduced vHIP-PoCG FC may represent a candidate neuroimaging correlate in MCID and may underlie APOE +4-related vulnerability to depressive and cognitive symptoms. Moreover, the mediating effect of vHIP-PoCG FC may partly account for the association among APOE +4 gene dose, depressive symptoms, and cognitive impairment in MCID.

## 1 Introduction

Mild cognitive impairment (MCI) is the symptomatic predementia phase of Alzheimer’s disease (AD) mainly marked by memory decline and cognitive impairment, commonly accompanied by neuropsychiatric symptoms (NPS) (Khachaturian, 2025; Albert et al., 2011). Subsyndromal depression (SSD) is one of the most common NPS in MCI, with an incidence rate ranging from 37% to 46% (Huang et al., 2024). MCI patients with SSD (MCID) exhibit poorer quality of life and greater impairments in daily activities (Mackin et al., 2013; Al Shamsi et al., 2024). Moreover, SSD in MCI has been associated with greater cognitive deficits, AD-related brain atrophy, faster cognitive decline, and increased risk of progression to dementia (Tan et al., 2019). Of note, compared with non-depressed MCI patients, MCID individuals exhibit more pronounced deficits in language memory, executive function, processing speed, and visual memory (Dong et al., 2016). These findings support that MCID is not simply a combination of MCI and depression but rather represents a distinct clinical phenotype with unique neural substrates. Therefore, elucidating the distinct clinical and neurobiological characteristics of MCID is essential for the development of targeted interventions aimed at addressing both cognitive and depressive symptoms.

Genetic factors, particularly the apolipoprotein E (APOE) ε4 allele, play a crucial role in MCI and AD pathogenesis. Notably, recent studies suggest that APOE ε4 homozygosity represents a genetic subtype with higher risk and potentially distinct neural mechanisms compared with heterozygotes or non-carriers (Fortea et al., 2024; Rajic Bumber et al., 2025). Previous studies have shown that individuals carrying two copies of APOE ε4 have a lifetime risk of developing AD is significantly higher than that of heterozygotes or non-carriers (Reas et al., 2024), reinforcing the key role of the APOE ε4 number in MCI. Accumulating evidence indicates that APOE ε4 is associated with accelerated cognitive decline and greater atrophy in hippocampal and neocortical structures, with some effects showing an allele dose-dependent pattern (Ma et al., 2022). APOE ε4 has also been associated with increased depressive symptom burden in older adults, suggesting that ε4-related vulnerability may extend from cognitive impairment to affective dysfunction (Lavretsky et al., 2003). However, most previous studies have classified individuals simply as APOE ε4 carriers or non-carriers, rather than explicitly modeling ε4 allele dosage. The neurobiological mechanisms linking APOE ε4 allele burden to the coexistence of cognitive impairment and depressive symptoms in MCID remain largely unclear.

With the rapid advancement of neuroimaging techniques, APOE ε4-related neural alterations in specific brain regions have been increasingly investigated (Chen et al., 2024). A previous meta-analysis found that hippocampal atrophy is significantly greater in APOE ε4 carriers compared with non-carriers (Belitskaya-Levy et al., 2018), and studies of geriatric depression report pronounced hippocampal volume reductions in APOE ε4 carriers, especially in the right hippocampus (Kim et al., 2002). Previous studies have found that APOE ε4 carriers showed increased connectivity between the left hippocampus and the left insula and lateral prefrontal region (De Marco et al., 2017), indicating that the APOE ε4 allele is associated with a more pronounced degree of hippocampal network breakdown. Although previous studies have examined APOE ε4-related alterations in hippocampal connectivity, the extent to which these alterations vary according to ε4 allele dosage in patients with MCID remains unclear. Therefore, the present study aimed to characterize connectivity alterations associated with APOE ε4 heterozygosity and homozygosity in MCID.

Anatomical and functional studies have established that the hippocampus exhibits distinct dorsal (dHIP) and ventral (vHIP) subregions, with the dHIP primarily involved in cognitive processing and the vHIP more closely linked to emotion regulation (Fanselow and Dong, 2010; Poppenk et al., 2013). Emerging evidence suggests that the vHIP may be particularly susceptible to APOE ε4-related alterations in stress- and emotion-related processes (Fang et al., 2021). The vHIP has distinct molecular and behavioral stress responsivity compared with the dHIP, which primarily supports cognitive processing, providing a functional basis for differential vulnerability (Floriou-Servou et al., 2018). Importantly, both the APOE ε4 genotype and elevated amyloid−β (Aβ) burden have been linked to alterations in large-scale functional brain networks, including disruption of default mode network organization and hippocampal–cortical interactions (Sheline et al., 2010; Song et al., 2025). APOE ε4 carriers show altered network connectivity patterns that are exacerbated in the presence of Aβ pathology, suggesting that amyloid accumulation contributes to functional network dysfunction in an APOE ε4-dependent manner. However, the allele dose-dependent effects of APOE ε4 on dHIP and vHIP connectivity in patients with MCID remain unclear. Therefore, the present study specifically aimed to examine these subregion-specific APOE ε4 allele-dosage effects and their associations with depressive symptoms and cognitive function in patients with MCID.

In this study, patients with MCID were stratified into three groups according to their APOE ε4 allele status: APOE ε4 non-carriers, APOE ε4 heterozygotes, and APOE ε4 homozygotes. We hypothesized that APOE ε4 homozygotes exhibit characteristic alterations in hippocampal subregional FC, which may mediate the relationships between APOE ε4 gene dosage and clinical manifestations in MCID patients. To test this hypothesis, we first performed FC analyses of the vHIP and dHIP across the three groups. Next, correlation analyses were conducted to assess the associations between abnormal hippocampal FC and clinical symptoms. Finally, mediation analyses were applied to determine whether hippocampal subregional FC mediated the associations of APOE ε4 status with depressive symptoms and cognitive function in MCID.

## 2 Materials and methods

### 2.1 Study participants

The data for this article were obtained from the Alzheimer’s Disease Neuroimaging Initiative (ADNI) database.^1^ The ADNI was launched in 2003 as a public-private partnership led by Principal Investigator Michael W. Weiner, MD. Subjects with MCI meet the criteria for MCI defined by the National Institute of Neurological and Communicative Disorders and Stroke-Alzheimer’s Disease and Related Disorders Association (NINCDS-ADRDA) (McKhann et al., 2011). The 15-item Geriatric Depression Scale (GDS-15) was used to assess depressive symptoms. In older adults, a GDS-15 score higher than 5 is commonly used as a screening threshold for clinically significant depressive symptoms. In the present study, SSD was defined as GDS-15 scores greater than 0 and less than 6. Thus, participants with GDS-15 scores ≥ 6 were not classified as having SSD. Accordingly, MCID was defined as MCI patients accompanied by SSD, assessed using the GDS-15 scores greater than 0 and less than 6, in accordance with established criteria from recent studies (Zhang et al., 2020). The use of ADNI data was approved by the institutional review boards of all participating institutions. Moreover, written informed consent was obtained from all participants or their guardians, in accordance with the Declaration of Helsinki. Detailed up-to-date information was obtained from the ADNI database.

### 2.2 Neuropsychological assessment

We used Mini-Mental State Examination (MMSE) scores to evaluate participants’ global cognition (Arevalo-Rodriguez et al., 2015). To assess cognitive function across diverse domains, we used the ADNI composite scores for memory (ADNI-MEM), executive functioning (ADNI-EF), language (ADNI-LAN), and visuospatial functioning (ADNI-VS) (Crane et al., 2012). In addition, the Alzheimer’s Disease Assessment Scale Cognitive subscale (ADAS-Cog), consisting of 13 items (ADAS-Cog13), was also used (Wessels et al., 2018). We used Geriatric Depression Scale (GDS) scores (Lopez et al., 2010) to measure depressive symptoms. In this study, neuropsychiatric symptoms were assessed by the neuropsychiatric inventory (NPI) scores (Sinclair et al., 2023).

### 2.3 APOE genotyping

APOE genotypes were determined using standard polymerase chain reaction methods, as previously described. To account for the protective effect of the APOE ε2 allele (Jackson et al., 2024), we excluded all subjects carrying the APOE ε2 allele. In this study, MCID patients were divided into three groups: APOE ε4 non-carriers, APOE ε4 homozygotes, and APOE ε4 heterozygotes.

### 2.4 Image acquisition and preprocessing

All MRI data in this article were acquired from the ADNI database using Philips scanners (Manufacturer = Philips Healthcare/Philips Medical Systems), standardized according to the ADNI protocol.^2^ All scans were obtained using a sagittal three-dimensional magnetization prepared rapid acquisition gradient-echo (MP-RAGE) sequence with a voxel size of 1 mm × 1 mm × 1.2 mm, and the raw DICOM images were obtained from the public ADNI site. For the functional images, the following parameters were used: TR = 3,000 ms, TE = 30 ms, flip angle = 80°, slice thickness = 3.3 mm, voxel size = 3.3 mm^3^ × 3.3 mm^3^ × 3.3 mm^3^, matrix = 64 × 64, and 140 time points in each run. Further protocol details are available on the official ADNI webpage.^3^

### 2.5 Functional connectivity analysis

Imaging preprocessing was performed using the Data Processing Assistant for Resting-State fMRI (DPARSF)^4^ (Yan et al., 2016). The first 10 images were discarded, and slice timing and head motion correction were applied to the remaining 130 images. To minimize the effect of head motion, we excluded the participants whose maximal head movement translation exceeded 2 mm, whose mean framewise displacement (FD) exceeded 0.2 mm, or whose rotation exceeded 2°. Next, the DARTEL algorithm was used to transform the images into standard Montreal Neurological Institute (MNI) space, and each voxel was spatially resampled to a 3 mm^3^ × 3 mm^3^ × 3 mm^3^ voxel size. The CSF signal, white matter, and Friston-24 motion parameters were considered nuisance covariates. Subsequently, functional images were spatially smoothed with a 6 mm full-width half-maximum (FWHM) isotropic Gaussian kernel. Bandpass filtering (0.01–0.08 Hz) was applied to decrease the effect of systematic drift and high-frequency noise. Considering the multi-center nature of the ADNI MRI data, scanner-related variables, including scanner site and MR acquisition information, were additionally included as covariates in the statistical models to minimize the potential influence of site- and scanner-related variability. Residual head motion was quantified using mean framewise displacement (FD), and mean FD was also included as an additional covariate to confirm the robustness of the results.

Four subregions in each hemisphere, comprising the dorsal hippocampus (dHIP) and ventral hippocampus (vHIP), were created using cytoarchitectonically defined probabilistic maps of the hippocampus (Strange et al., 2014). Maximum probability maps were used to create nonoverlapping hippocampus subregions using the SPM Anatomy Toolbox. We conducted seed-based rs-FC analysis using the Resting-State fMRI Data Analysis Toolkit (REST).^5^ First, Pearson’s correlation coefficients were calculated between the mean time course of each seed and voxel in the whole brain. Next, voxel-wise correlation analyses were performed between each seed and the remaining brain voxels to obtain FC maps. Fisher’s Z transformation standardized Pearson’s correlation coefficients. Then, group comparisons of the Z-value maps of the bilateral vHIP and dHIP were performed, with age, sex, and education as covariates. A Gaussian random field (GRF) correction was applied for multiple-comparison correction with a voxel-level threshold of *p* < 0.001. To account for the four hippocampal seed-based analyses, the cluster-level significance threshold was further Bonferroni-corrected by dividing 0.05 by 4, resulting in a corrected cluster-level threshold of *p* < 0.0125, two-tailed.

### 2.6 Statistical analysis

We tested all data for normality using the Shapiro–Wilk test. For data following a normal distribution, we assessed differences in demographic and clinical profiles using ANCOVA. We conducted *post-hoc* tests for any two groups, and used χ^2^ tests for gender. To identify differences among the means of the three groups, we used Fisher’s least significant difference (LSD) test with age, gender, and education as covariates. For data that did not follow a normal distribution, we compared differences among the three groups using the nonparametric Kruskal–Wallis test. To further examine the dose-dependent effect of APOE ε4, we performed a linear regression analysis with APOE ε4 allele dose coded as an ordinal variable according to the number of ε4 alleles (0 = ε3/ε3, 1 = ε3/ε4, 2 = ε4/ε4).

Multicollinearity was assessed using tolerance, variance inflation factor, and Pearson’s correlation coefficients. We then used SPSS PROCESS (Hayes, 2013) to conduct mediation analyses to examine whether hippocampal subregional FC mediated the relationships among APOE ε4 gene dose, depressive symptoms and cognitive function in MCID, controlling for age, gender, and education. Bootstrapping analysis with 10,000 resamples calculated the 95% confidence interval (CI) for the mediation effects in MCID. Bootstrapped 95% CI, not including zero, were considered significant.

## 3 Results

### 3.1 Demographic and clinical characteristics

In this study, 88 patients with MCID were divided into 31 APOE ε4 heterozygotes, 12 APOE ε4 homozygotes, and 45 non-carriers. The demographic and clinical information is summarized in Table 1. We found no significant differences in age, gender, or education among APOE ε4 homozygotes, heterozygotes, and non-carriers. The MMSE score was significantly lower in the APOE ε4 homozygotes compared to non-carriers (*p* = 0.021). The ADAS-Cog13 score (*p* = 0.017) and ADNI-LAN score (*p* = 0.025) were significantly higher in the APOE ε4 homozygotes compared to non-carriers. There were no significant differences in the ADNI-MEM, ADNI-EF, ADNI-VS, GDS-15 and NPI scores among the three groups (Table 1).

**TABLE 1:** Demographic data and clinical measures among MCID patients with APOE ε4 heterozygotes, APOE ε4 homozygotes, and non-carriers.

Groups	APOE e4(−/−)	APOE e4(+/−)	APOE e4(+/+)	*F*-value	*p*-value
Subjects	45	31	12	–	–
Age	76.34 ± 8.57	75.92 ± 6.87	74.06 ± 6.93	0.41	0.66
Sex (F/M)	25/20	17/14	7/5	0.04	0.97
Education	12.51 ± 2.71	12.19 ± 2.90	12.04 ± 3.19	0.19	0.83
MMSE	23.32 ± 1.70	22.87 ± 1.59	22.00 ± 1.63	3.32	< 0.05^a^
ADAS-Cog13	30.03 ± 9.15	33.88 ± 9.44	37.36 ± 9.23	3.58	< 0.05^a^
ADNI-MEM	−0.98 ± 0.59	−0.99 ± 0.57	−0.91 ± 0.56	0.09	0.92
ADNI-EF	−1.19 ± 0.87	−1.16 ± 0.90	−1.09 ± 1.05	0.06	0.94
ADNI-LAN	−1.46 ± 1.17	−0.99 ± 0.98	−0.67 ± 0.82	3.42	< 0.05^a^
ADNI-VS	−0.63 ± 0.95	−0.77 ± 1.10	−0.72 ± 1.04	0.18	0.84
GDS-15	2.39 ± 1.42	2.03 ± 1.25	2.28 ± 1.55	0.70	0.51
NPI	2.15 ± 1.28	2.16 ± 1.69	2.62 ± 1.94	0.48	0.62

MMSE, Mini-Mental State Examination; GDS, Geriatric Depression Screening Scale; ADAS-Cog, Alzheimer’s Disease Assessment Scale Cognitive subscale; ADNI-MEM, ADNI composite scores for memory; ADNI-EF, ADNI composite scores for executive function; ADNI-LAN, ADNI composite scores for language; ADNI-VS, ADNI composite scores for visuospatial function.

*^a^*APOE e4(−/−) vs. APOE e4(+/+),

*^b^*APOE e4(−/−) vs. APOE e4(+/−), and

*^c^*APOE e4(+/−) vs. APOE e4(−/−).

### 3.2 Hippocampal subregion FC analysis results

**FIGURE 1:**
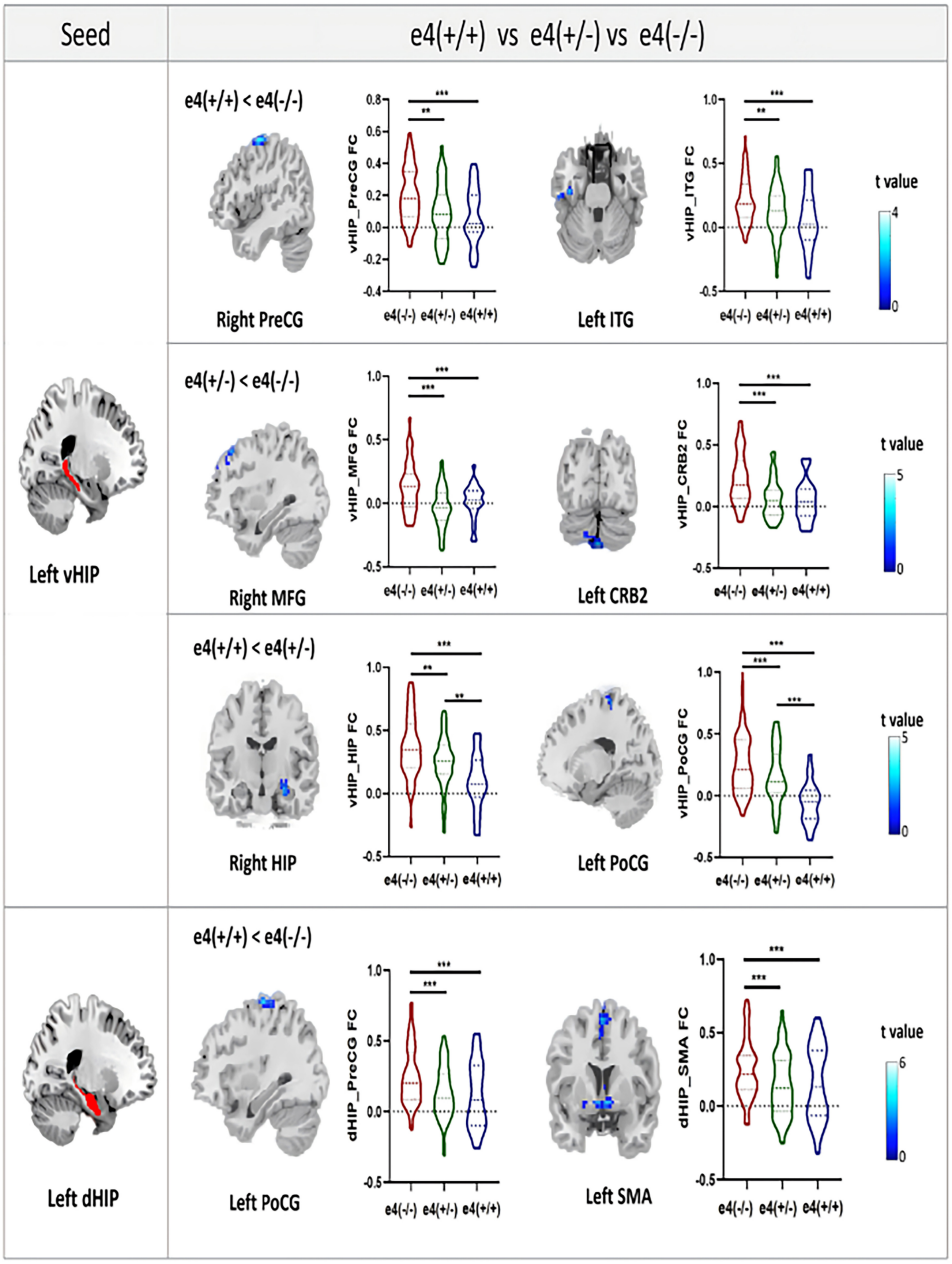
Group differences in seed-based functional connectivity between bilateral hippocampal subregions and the whole brain in MCID patients with one ε4 allele, two ε4 alleles, and non-carriers (voxel-level *p* < 0.001, a Bonferroni-corrected cluster-level threshold of *p* < 0.0125, two-tailed). The color bar represents the range of T values. dHIP, dorsal hippocampus; vHIP, ventral hippocampus; PreCG, precentral gyrus; ITG, inferior temporal gyrus; MFG, middle frontal gyrus; CRB2, Cerebellum_Crus2; PoCG, postcentral gyrus; SMA, supplementary motor area. (*p < 0.05; **p < 0.01; ***p < 0.001).

Compared to MCID patients with non-carriers, we found decreased FC between the left vHIP and left inferior temporal gyrus (ITG), and right precentral gyrus (PreCG) in APOE ε4 homozygotes. Compared to MCID patients with non-carriers, we found decreased FC between the left vHIP and the right middle frontal gyrus (MFG) and the left Cerebellum_Crus2 (CRB2) in APOE ε4 heterozygotes. Moreover, we found decreased FC between the vHIP and the left postcentral gyrus (PoCG) and the right HIP in MCID patients with APOE ε4 homozygotes compared to APOE ε4 heterozygotes. Compared to MCID patients with non-carriers, we found decreased FC between the left dHIP and the left PoCG, and the left supplementary motor area (SMA) in APOE ε4 homozygotes (Figure 1 and Table 2). However, there was no difference in FC of the right vHIP and the right dHIP among the three groups.

**TABLE 2:** Group differences in whole-brain functional connectivity of the bilateral vHIP and dHIP among MCID patients with one ε4 allele, two ε4 alleles, and non-carriers.

	Cluster location	MNI coordinates (x, y, z)	Cluster size	T-value
**Seed: left vHIP**				
APOE e4(+/+) < e4(−/−)				
	Right precentral gyrus	45, −9, 60	758	−5.63
	Left inferior temporal gyrus	−39, −18, −27	47	−5.15
APOE e4(+/−) < e4(−/−)				
	Left Cerebellum_Crus2	0, −81, −45	83	−4.75
	Right middle frontal gyrus	33, 30, 48	61	−4.56
APOE e4(+/+) < e4(+/−)				
	Right hippocampus	27, −12, −15	48	−4.09
	Left postcentral gyrus	−18, −30, 69	48	−3.98
**Seed: left dHIP**				
APOE e4(+/+) < e4(−/−)				
	Left postcentral gyrus	−39, −30, 54	221	−5.29
	Left supplementary motor area	0, 6, 60	101	−4.93

### 3.3 Correlation analysis results

**FIGURE 2:**
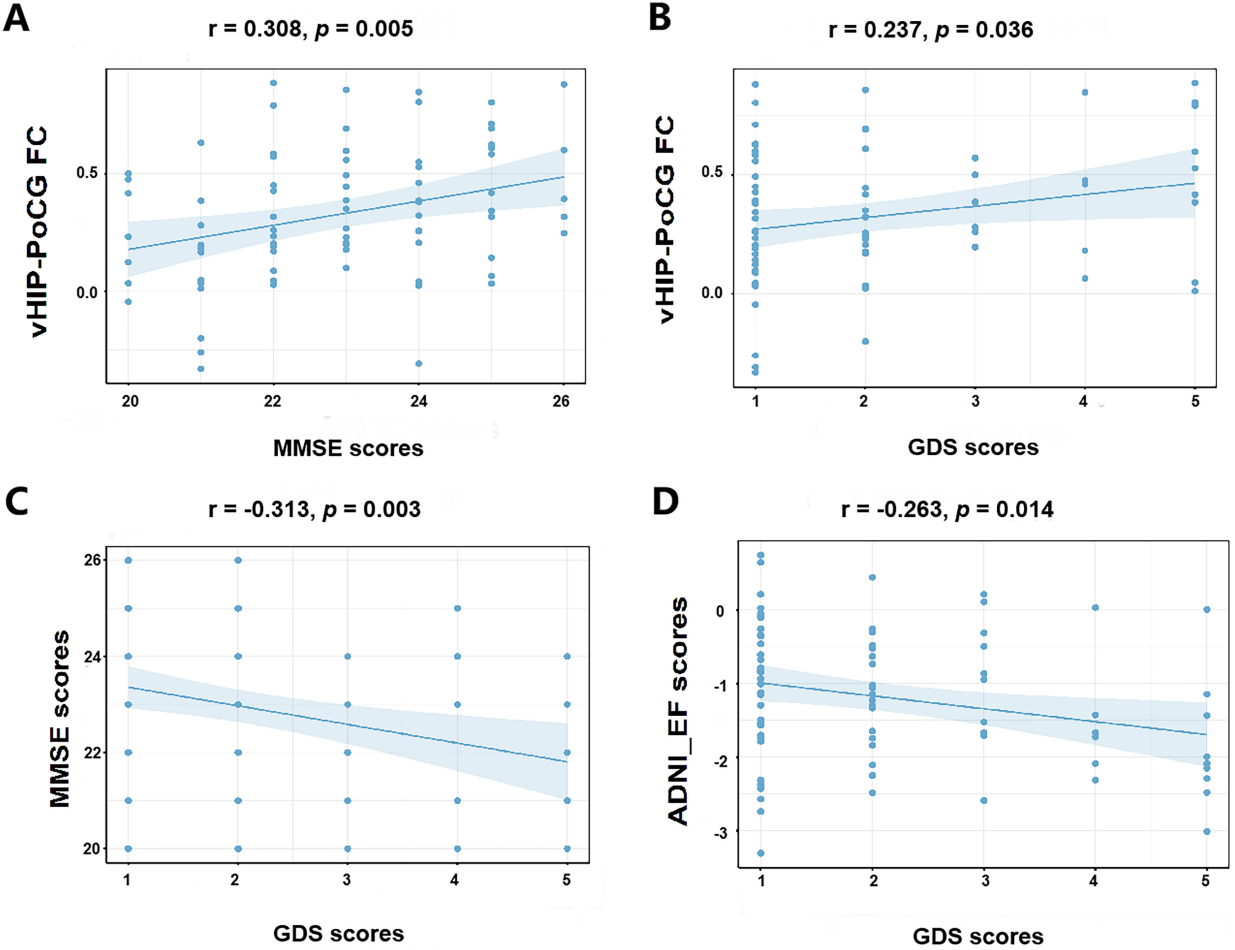
Associations between vHIP-PoCG FC and clinical measures in MCID. Scatter plots show the relationships between Fisher z-transformed FC of the vHIP-PoCG FC and MMSE **(A)**, GDS-15 **(B)**, and ADNI-EF scores **(C)**, as well as the relationship between ADNI-EF scores and GDS scores **(D)**. Associations were examined using partial correlation analyses controlling for age, sex, education, and mean framewise displacement. MMSE, Mini-Mental State Examination; GDS-15, 15-item Geriatric Depression Scale; ADNI-EF, Alzheimer’s Disease Neuroimaging Initiative executive function composite score.

In the MCID group, FC between the vHIP and PoCG showed significant positive correlations with both MMSE scores (*r* = 0.308, *p* = 0.005) and GDS scores (*r* = 0.237, *p* = 0.036) (Figures 2A, B). Moreover, GDS scores were significantly negatively correlated with MMSE scores (*r* = −0.313, *p* = 0.003) and ADNI_EF scores (*r* = −0.263, *p* = 0.014) (Figures 2C, D). To further clarify whether the association between vHIP-PoCG FC and GDS scores differed across APOE genotype groups, we performed genotype-stratified correlation analyses. The positive correlation was significant in the APOE ε4/ε4 group (*r* = 0.693, *p* = 0.009), whereas no significant correlation was observed in the ε3/ε4 group (*r* = 0.241, *p* = 0.268) or the ε3/ε3 group (*r* = 0.005, *p* = 0.977) (Supplementary Figure 1). Linear regression analysis revealed a significant negative association between APOE ε4 allele dose and vHIP-PoCG FC. Specifically, each additional ε4 allele was associated with lower vHIP-PoCG FC (β = –0.118, 95% CI: −0.197 to −0.039). Scatter plot visualization showed a dose-dependent decline in vHIP-PoCG FC across ε3/ε3, ε3/ε4, and ε4/ε4 groups, consistent with the regression trend (Supplementary Figure 2).

### 3.4 Mediation analysis results

**FIGURE 3:**
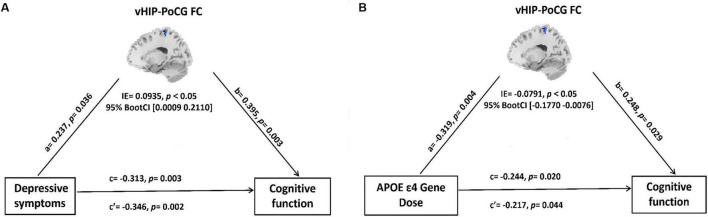
The mediation effect of FC of the vHIP and PoCG on the relationship of bilateral depressive symptom measured by GDS-15 scores and cognitive function measured by MMSE scores in MCID **(A)**; The mediation effect of FC of the vHIP and PoCG on the relationship between APOE ε4 gene dose and cognitive function in MCID **(B)**.

We found that FC between the vHIP and the left PoCG mediated the relationship between depressive symptoms measured by GDS-15 scores and cognitive function measured by MMSE scores (indirect effect = 0.0935, bootstrapped 95% CI = [0.0009, 0.2110], *p* < 0.05) in MCID (Figure 3A). We also found that FC between the vHIP and the left PoCG mediated the relationship between APOE ε4 gene dose and cognitive function measured by MMSE scores (indirect effect = −0.0791, bootstrapped 95% CI = [−0.1770, −0.0076], *p* < 0.05) in MCID (Figure 3B).

## 4 Discussion

Our study observed that the APOE ε4 homozygotes showed decreased FC of the left vHIP and left PoCG, and FC of left vHIP and right HIP compared to MCID patients with APOE4 heterozygotes. Additionally, FC between the vHIP and the left PoCG mediated the association between depressive symptoms and cognitive function, as well as the association between APOE ε4 gene dose and cognitive function in MCID. These findings suggest that vHIP-PoCG connectivity may partly account for the association between depressive symptoms, APOE ε4 gene dose, and cognitive function in MCID. Reduced hippocampal subregional connectivity, particularly between the vHIP and PoCG, may therefore represent a candidate neuroimaging correlate of cognitive impairment in MCID.

### 4.1 FC alteration in ε4 homozygotes vs. non-carriers

We observed that, compared with non-carriers, MCID patients with APOE ε4 homozygosity exhibited decreased FC between the left vHIP and the right PreCG, as well as between the left vHIP and the left ITG. In addition, MCID patients with APOE ε4 heterozygosity showed decreased FC between the right vHIP and the right MFG. The PreCG is a major component of the sensorimotor network (SMN), which supports the integration of sensory and motor processing (Yeo et al., 2011; Caspers et al., 2021). Consistent with our findings, previous studies have shown that APOE ε4 is associated with altered intrinsic network connectivity and poorer cognitive performance (Machulda et al., 2011; Cacciaglia et al., 2023). The ITG is involved in semantic processing, object recognition, and higher-order integration, and also interacts with the default mode network (DMN) (Yu et al., 2025). Prior work has suggested that APOE ε4 is associated with abnormal DMN-related functional organization (Filippini et al., 2009; Song et al., 2025), which may in turn affect ITG-related processing. The MFG is a key region within the central executive network (CEN) and is involved in executive control, attention regulation, and the integration of memory-related information (Ma et al., 2025). Previous studies have further shown that APOE ε4 is associated with structural and functional alterations in frontal regions, including the MFG (Memel et al., 2021), which may contribute to memory-related impairment. Taken together, our findings suggest that APOE ε4 gene dose may be associated with alterations in vHIP FC involving sensorimotor, temporal, and prefrontal cortical regions, providing preliminary evidence for network-level changes in MCID.

### 4.2 FC alteration in ε4 homozygotes vs. heterozygotes

We observed decreased FC between the left vHIP and the left PoCG, as well as between the left vHIP and the right HIP in MCID patients with APOE ε4 homozygosity compared to heterozygosity. The PoCG, along with the PreCG, forms part of the sensorimotor network (SMN), which integrates sensory and motor processes essential for cognitive resilience (Zhou et al., 2022). Disruption of the SMN, including altered PoCG connectivity, has been implicated in cognitive decline in neurodegenerative diseases (Ossenkoppele et al., 2020). Homozygous carriers of APOE ε4 exhibited a faster rate of memory decline compared with non-carriers (Lim et al., 2018), suggesting a cumulatively detrimental effect of APOE ε4 on cognitive decline. APOE ε4 may accelerate clinical decline by compromising large-scale brain network integrity and disrupting functional connectivity, as evidenced by a faster rate of cognitive decline in ε4 homozygotes and altered resting-state functional network organization in APOE ε4 carriers relative to non-carriers. Moreover, the genotype-stratified correlation analysis provided additional insight into the relationship between vHIP-PoCG FC and depressive symptoms. The positive correlation between vHIP-PoCG FC and GDS scores was primarily observed within the ε4/ε4 group. The stronger positive association in ε4/ε4 carriers may suggest that hippocampal connectivity is more closely linked to depressive symptom in patients with higher APOE-related genetic risk. One possible explanation is that increased vHIP-PoCG coupling among ε4/ε4 carriers may represent a distinct connectivity pattern related to greater depressive symptom. However, given the exploration of the subgroup analysis and the limited subgroup sample size, the findings should be considered preliminary and requires validation in larger independent cohorts.

### 4.3 vHIP-specific connectivity alteration pattern

Our results showed a selective reduction in vHIP connectivity, rather than dHIP connectivity, in APOE ε4 homozygotes, suggesting that the vHIP may be more vulnerable during the early stages of cognitive impairment, particularly in individuals with a greater APOE ε4 allele load. The vHIP preferentially subserves emotional and contextual processing, whereas the dHIP is more strongly linked to spatial memory and cognitive functions, consistent with established dorsal-ventral hippocampal specialization (Plachti et al., 2019; Adnan et al., 2016). Furthermore, previous studies have reported altered resting-state FC involving the ventral hippocampus and large-scale networks such as the DMN and salience network in APOE ε4 carriers (Wang et al., 2015; Seoane et al., 2024), supporting the notion that vHIP disruption contributes to network dysfunction in cognitive decline. In line with these observations, our findings suggest that altered connectivity between the vHIP and the PoCG may reflect APOE ε4-related network dysfunction in MCID. Taken together, these results suggest that FC between the left vHIP and the left PoCG may represent a candidate neuroimaging correlate of MCID-related alterations, with this association appearing more pronounced in individuals with APOE ε4 homozygosity.

### 4.4 The mediating effect of vHIP-PoCG FC

We found that FC between the vHIP and the left PoCG mediated the relationship of depressive symptoms on cognitive function, as well as the relationship between APOE ε4 gene dose and cognitive function in MCID. Prior work found that hippocampal-based FC mediated the relationship between depressive symptoms and cognitive function, with a mediating effect of 48% (Slot et al., 2019). Our findings of the mediating effect of vHIP-PoCG FC on the relationship between depression and cognition indicated that higher depressive symptoms were associated with increased vHIP-PoCG FC, which in turn was positively associated with cognitive performance. However, the indirect association through vHIP-PoCG FC was in the opposite direction to the total association between depressive symptoms and cognition. This pattern is more consistent with an inconsistent mediation or suppressor-like effect than with a conventional mediation pathway, implying that vHIP-PoCG connectivity may partially counteract the detrimental impact of depressive symptoms on cognition. Furthermore, evidence from previous studies indicates that the dHIP might be particularly vulnerable to early modulation in APOE ε4 carriers (Stocker et al., 2021; Zheng et al., 2018), which may help explain the differing mechanisms of gene-modulated cognitive dysfunction in MCID. Our previous studies found that hippocampal FC moderated the relationship between hippocampal volume and cognitive function in MCID (Du et al., 2022, 2024), suggesting that APOE ε4 allele load could either exacerbate or ameliorate the effects of hippocampal FC on cognitive function. In our cross-sectional study, FC between the left vHIP and left PoCG was associated with cognitive function in MCID, suggesting that this connectivity pattern may represent a candidate neural correlate of cognitive performance in this population. These findings provide a basis for further investigation into how APOE ε4-related connectivity alterations relate to cognitive outcomes in MCID.

### 4.5 Limitations

This study has several limitations. First, the cross-sectional design limits our ability to establish the predictive value of the APOE genotype for MCID progression. Longitudinal studies are needed to better understand the relationship between changes in neural biomarkers and cognitive function in MCID, and to clarify the underlying mechanisms. Second, the sample size of APOE ε4 homozygotes was relatively small, reflecting the low prevalence of the ε4/ε4 genotype in the general population. This limited subgroup size reduced statistical power and may have affected the stability and generalizability of findings derived from subgroup comparisons. Although the ordinal allele-dose analysis provided complementary evidence for a dose-dependent association between APOE ε4 burden and vHIP-PoCG FC, conclusions regarding the ε4/ε4 subgroup should be considered preliminary and exploratory and require validation in larger, independent cohorts. Third, the definition of subthreshold depression remains poorly standardized. MCID was defined as MCI accompanied by SSD, with GDS-15 scores greater than 0 and less than 6 in this study. Although this definition is consistent with previous studies, the relatively low threshold may have included individuals with very mild or transient mood symptoms. Therefore, the current findings should be interpreted as neural correlates of SSD in MCI rather than definitive neural mechanisms of clinically depression. Future research should aim for a more precise and universally accepted definition to facilitate the recruitment of more homogeneous samples and to better characterize the behavioral and neurobiological features of SSD in MCI. Finally, the mediation analyses were based on cross-sectional data, which precludes causal inference regarding the temporal or mechanistic relationships among APOE ε4 allele dose, vHIP-PoCG connectivity, depressive symptoms, and cognitive performance. Longitudinal studies with larger samples and independent cohort validation are needed to confirm the robustness, temporal directionality, and clinical relevance of these associations.

In conclusion, MCID patients with APOE ε4 homozygosity showed reduced functional connectivity between the vHIP and the left PoCG, as well as between the left vHIP and the right hippocampus, compared with APOE ε4 heterozygotes. These results suggest that APOE ε4 gene dose may be associated with hippocampal subregional connectivity alterations in MCID. Mediation analysis further indicated that vHIP-PoCG connectivity may partly account for the associations of APOE ε4 gene dose and depressive symptoms with cognitive function. These findings provide preliminary evidence that vHIP-PoCG connectivity is involved in MCID-related neural alterations and warrants further investigation in longitudinal and interventional studies.

## Data Availability

The datasets presented in this study can be found in online repositories. The names of the repository/repositories and accession number(s) can be found in this article/Supplementary material.

## Footnotes

1
https://adni.loni.usc.edu/

2
http://adni.loni.usc.edu/methods/documents/mri-protocols/

3
http://adni.loni.usc.edu/methods/documents/

4
http://www.restfmri.net/forum/dparsf

5
http://resting-fmri.sourceforge.net

## Supplementary material

Supplementary Figure 1 The genotype-stratified correlation between vHIP-PoCG FC and GDS scores in the APOE ε3/ε3, ε3/ε4, and ε4/ε4 groups.

Supplementary Figure 2 Dose-dependent association between APOE ε4 allele dose and vHIP–PoCG FC. The scatter plot shows a graded decline in vHIP–PoCG FC across APOE ε3/ε3, ε3/ε4, and ε4/ε4 groups.
